# Mastectomy Reconstruction Techniques for Gender Diverse Breast Cancer and High Risk Patients: A Case Series and Literature Overview

**DOI:** 10.3390/jcm15020441

**Published:** 2026-01-06

**Authors:** Thais Calderon, James T. Antongiovanni, Danielle J. Eble, Alisha L. Nguyen, Chizoba A. Mosieri, Andreea Gavrilescu, Sarah R. Goldsberry-Long, Rachel B. Lentz, Suzanne M. Inchauste

**Affiliations:** 1 Division of Plastic Surgery, Department of Surgery, University of Washington, Seattle, WA 98195, USArblentz@uw.edu (R.B.L.); 2 Elson S. Floyd College of Medicine, Washington State University, Spokane, WA 99164, USA; 3 School of Medicine, Louisiana State University Health Sciences Center Shreveport, Shreveport, LA 71103, USA; 4 School of Medicine, University of Washington, Seattle, WA 98195, USA; 5 Charleston Area Medical Center, Charleston, WV 25301, USA; 6 Division of Plastic and Reconstructive Surgery, University of Wisconsin, Madison, WI 53706, USA

**Keywords:** gender-affirming breast reconstruction, oncologic gender-affirming mastectomy, non-binary reconstruction, breast reconstruction techniques

## Abstract

**Background/Objectives**: Assigned female at birth (AFAB) individuals who identify as transgender or gender-diverse (TGD) with concurrent breast cancer or high-risk genetic mutations represent a unique population, requiring consideration of oncologic and aesthetic goals. These patients sought chest masculinization with oncologic gender-affirming mastectomy (OGAM) or non-binary reconstruction to alleviate gender dysphoria and treat their breast cancer. There is limited literature on surgical techniques in this patient population. **Methods**: A retrospective chart review of AFAB TGD adults (>18 years of age) who underwent OGAM or non-binary reconstruction at the University of Washington between 2019 and 2023 was conducted. All patients had a consultation with a plastic surgeon for reconstruction and a minimum of one year follow-up. Demographic data, oncologic status, post-operative complications, and revision surgical history were collected. **Results**: Eight AFAB TGD individuals met the inclusion criteria. The mean age at the time of mastectomy was 35.13 years (SD = 8.04), and the mean BMI was 29.88 (SD = 6.40). Indications for mastectomy included a breast cancer diagnosis (*N* = 4) or a strong family history of breast cancer or genetic predisposition (*N* = 4). Two (25%) patients underwent nipple-sparing mastectomies (NSM), two patients (25%) underwent skin-sparing mastectomy with Goldilocks reconstruction, and four patients (50%) underwent simple mastectomy (oncologic gender-affirming mastectomy), flat closure with free nipple graft (FNG). Two patients had staged nipple mastectomy with secondary nipple reduction and fat grafting. Six patients had immediate reconstruction, four (50%) patients underwent immediate double-incision OGAM with FNG, and two (25%) patients underwent Goldilocks procedures—one with and one without FNG. One patient (12.5%) experienced a surgical site infection, and three patients (37.5%) underwent revision surgery. No patients had positive margins following their mastectomy. **Conclusions**: This case series highlights the importance of a multidisciplinary and highly personalized approach for AFAB and TGD individuals undergoing oncologic gender-affirming mastectomy or non-binary reconstruction. We reviewed reconstructive options performed at our institution, demonstrating safe oncologic and reconstructive techniques that emphasized collaboration between breast and plastic surgeons.

## 1. Introduction

Breast cancer in the United States accounts for approximately 30% of all newly diagnosed cancers in women and individuals assigned female at birth (AFAB) [1,2,3]. The majority of the literature on breast cancer treatment and reconstruction is focused on (1) cisgender female patients and (2) implant-based or autologous reconstruction. However, it is estimated that 1.3 million individuals in the U.S. identify as transgender or gender diverse (TGD), with approximately 822,000 individuals identifying as either transgender male or gender diverse [4]. It is important to acknowledge that the risk of breast cancer amongst TGD individuals may approximate that of cisgender women, yet there is a paucity in the literature regarding TGD oncologic reconstruction [1].

Our breast health team at the University of Washington comprises various medical and surgical specialists with the aim of providing holistic and patient-centered care. It is standard practice that high-risk or newly diagnosed breast cancer patients are routinely referred to our plastic surgeons to discuss reconstruction options prior to undergoing oncologic resection. The current literature highlights the importance of multidisciplinary teams for breast cancer care and various breast reconstruction options, ranging from breast conservation with oncoplastic breast reduction to mastectomy with autologous free flap reconstruction in the cisgender female population [5,6,7,8]. However, there is limited literature describing procedures offered to TGD patients undergoing oncologic gender-affirming mastectomy (OGAM) or non-binary reconstruction [1]. To date, few publications have reported on simultaneous or staged OGAM and the various reconstructive surgical techniques available. One is a case series involving five patients, while another reviews a single case of OGAM. Both articles employed a double-incision approach with or without free nipple grafting (FNG) [1,9]. At our institution, our senior authors have combined a variety of reconstructive techniques for TGD OGAM patients, including double-incision mastectomy with FNG, nipple reduction and fat grafting, Goldilocks reconstruction with or without FNG, liposuction and fat grafting for chest contouring, and excision of excess lateral chest skin and tissue with various mastectomy approaches performed by the breast surgical oncologist. These techniques are employed in either a concurrent or staged fashion. We aim to contribute to the literature by discussing the diverse surgical techniques offered to our TGD patients along with their post-operative outcomes, highlighting safety and a variety of options for reconstruction and timing that are not currently reported in the literature.

## 2. Materials and Methods

A single institution retrospective chart review was conducted at the University of Washington, Seattle, WA, USA to identify AFAB patients with gender dysphoria and a concurrent diagnosis of (1) breast cancer or a (2) high-risk genetic mutation for breast cancer who underwent oncologic gender-affirming mastectomy and reconstruction between 2019 and 2023. English-speaking individuals who were 18 years of age or older with at least one year of follow-up from their reconstructive surgery were included. Patient demographics, past medical history, social history, oncologic status, hormonal therapy exposure, and perioperative data were collected from the electronic medical record. Perioperative data included type of mastectomy (nipple-sparing mastectomy (NSM), skin-sparing mastectomy (SSM), and simple mastectomy), staged versus immediate reconstruction, type of reconstruction technique used (double-incision gender-affirming mastectomy with free nipple grafting, Goldilocks reconstruction, nipple reduction, excess tissue resection, liposuction, and fat grafting), resection weights, complications, and revisions. Descriptive statistics were used to analyze categorical data. This study was determined to be exempt by our institutional review board (IRB STUDY0016745). All patients provided photographic consent for pre-, intra-, and post-operative images to be utilized for educational and scientific purposes, including publication.

### 2.1. Techniques

#### 2.1.1. Double-Incision Gender-Affirming Mastectomy with Free Nipple Grafting

**Figure 1:**
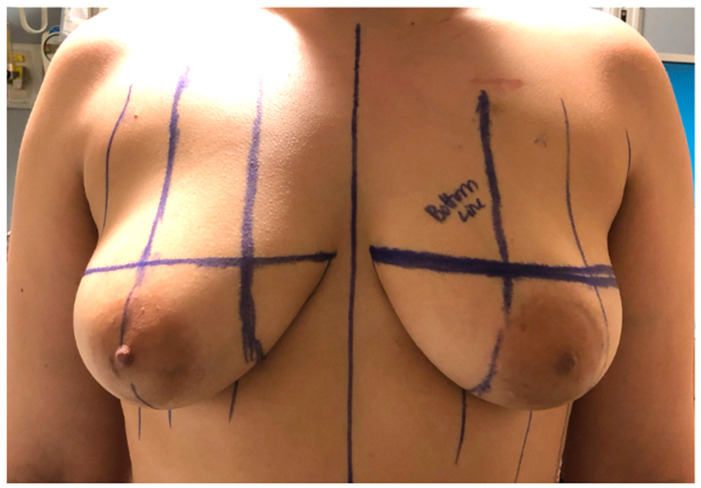
Markings for a double-incision gender-affirming oncologic mastectomy. Left breast comment “bottom line” indicates incision on the lower of the two markings.

The majority of patients underwent oncologic mastectomy (simple mastectomy) with transverse incisions and flat closure with FNG. Two patients underwent revision surgery with liposuction of the lateral chest wall and fat grafting to the chest to improve their overall contour. Simple mastectomies are commonly carried out for oncological care; however, for chest masculinization purposes, double-incision gender-affirming mastectomy markings are slightly modified to hide the final incision in the shadow of the inferior border of the pectoralis muscle. This is in contrast to the standard simple mastectomy with a transverse incision that is positioned in the middle of the breast with preservation of the inframammary fold (IMF) for possible delayed breast reconstruction. To ensure adequate length of the superior mastectomy flaps to reach the inferior border of the pectoralis muscle, patient markings are collaboratively performed by both the breast and plastic surgeon in the pre-operative area (Figure 1).

**Figure 2:**
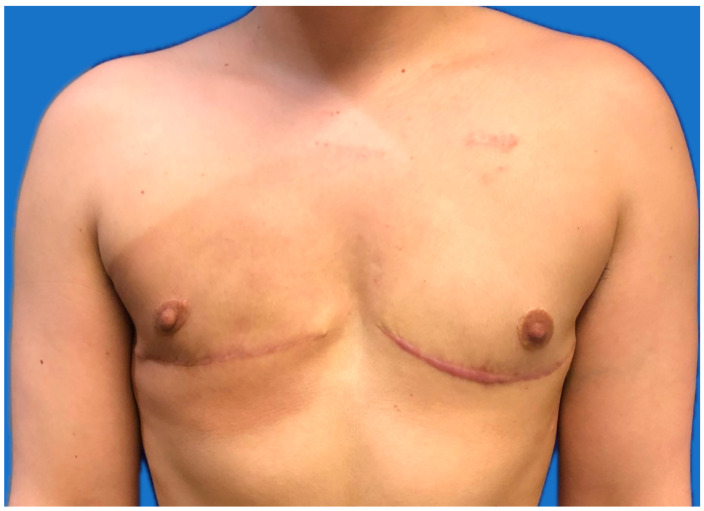
One year post-operative from double-incision oncologic gender-affirming mastectomy with free nipple graft with right chest post-mastectomy radiation.

The patient is brought to the operating theater when they undergo general anesthesia. Once the patient is intubated and their airway is secure, the patient is appropriately positioned supine with their arms padded and secured to arm boards, or one arm may be prepped into the field and secured on the field if sentinel lymph node mapping and biopsy are indicated. A sit test is performed with anesthesia to ensure there is sufficient length in the airway tubing if FNG is to be carried out, and the patient is prepped and draped in a sterile fashion. The plastic surgery team initiates the case by using a 34 mm or smaller nipple sizer to template the planned free nipple grafts. The nipples are scored with a 15-blade to the level of the superficial dermis. The breast team then proceeds with their oncologic mastectomy utilizing the marked superior incision to elevate the superior mastectomy flaps. The breast parenchyma is dissected with a combination of electrocautery and sharp dissection. The posterior aspect of the breast is elevated off the pectoralis muscle, intentionally elevating the pectoralis fascia with the breast parenchyma and clipping any perforating vessels. Once the breast is fully elevated down to the level of the inframammary fold, the plastic surgery team completes nipple graft harvest via sharp excision with a 10-blade. The free nipple grafts are then thinned and defatted on the back table with a curved iris scissor and stored in a wet 4 × 8 gauze to prevent the nipple grafts from drying out while the breast surgeon completes the mastectomies. The superior mastectomy flaps are pulled down over the chest to confirm the appropriate location of the inferior markings to allow for a flat closure without excess tension. Once this is confirmed bilaterally, the inferior incisions are made with a 10-blade and the mastectomy is completed. Obliteration of the IMFs is performed by the plastic surgery team to ensure a smooth transition from the chest wall to the abdomen. The breasts are marked for orientation and sent off to pathology with any additional margins as the breast team deems necessary. If indicated, the breast team proceeds with the sentinel lymph node biopsy at this time prior to completion of reconstruction. The plastic surgery team then begins with copious irrigation and hemostasis of the breast pocket. Bilateral 15 French drains are placed through lateral stab incisions to prevent seroma formation and to aid in adequate soft tissue healing to the pectoralis muscle. The superior and inferior mastectomy flaps are stapled together to ensure an appropriate flat closure and followed by a multilayer fashion with absorbable suture. If the patient is undergoing FNG, they are sat up, and nipple templates are placed to confirm symmetry and placement. It is important to note that a stereotypical cis-male chest has smaller, slightly oval-shaped nipples in a more lateral position compared to the larger, round-shaped and centrally located nipples of a stereotypical cis-female breast. It is important to discuss nipple position, size, and shape preferences with the patient prior to surgery. Once nipple placement is confirmed, the skin is de-epithelialized, hemostasis is achieved, and the nipples are sewn on with absorbable suture. Nipple bolsters are placed and secured with silk suture to prevent sheering and provide compression for optimal graft take. At the end of the case, the drains are put to bulb suction, and steri-strips are placed over the bilateral incisions, followed by placement of abdominal pads placed over the chest wall, and an ace bandage wrapped around the patient’s chest to provide constant compression. The standard result is a double incision with smaller, elliptical, laterally positioned nipples and an incision that mimics the curvature of the inferior border of the pectoral muscle visible in a cis-male chest (Figure 2).

#### 2.1.2. Free Nipple-Grafting and Nipple Reduction

**Figure 3:**
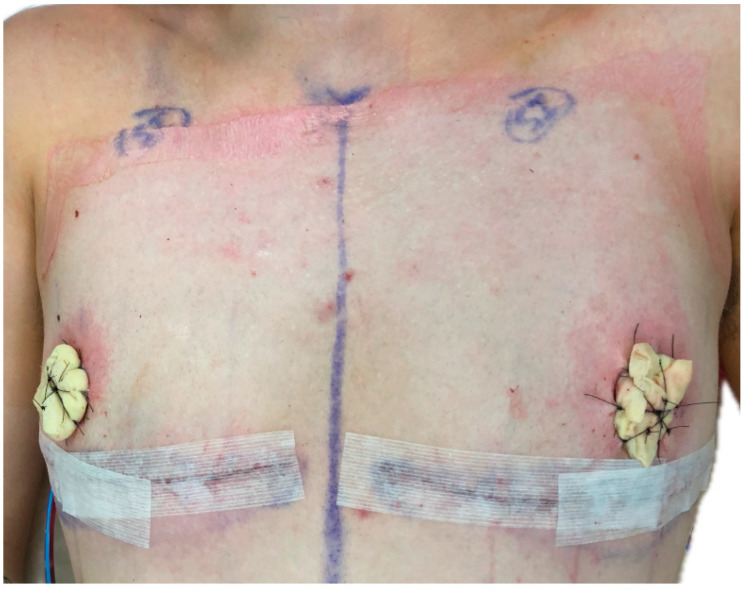
Intraoperative gender-affirming mastectomy with free nipple graft and tie-over nipple bolster.

In a traditional gender-affirming mastectomy with free nipple grafting, the native nipple is reduced to an oval shape, commonly 3 × 2 cm in dimension, and raised as a full-thickness skin graft. In excising the subcutaneous tissue, there is a significant reduction in the papilla height and size. Once the chest contour is complete, the patient is sat up in the operating room, and nipple templates are placed bilaterally to confirm the appropriate location. The templates are commonly placed 1.5 cm above the inferior incision line and 1.5 cm medial to the lateral border of the pectoralis muscle. Once confirmed, this area is de-epithelialized and prepared for dermal graft take. To ensure adequate incorporation, the nipples are sutured in place, followed by placement and suturing of nipple tie over bolster with petroleum gauze and cotton balls soaked with mineral oil (Figure 3). Nipple bolsters remain on for 5–7 days to ensure the dermal graft takes.

Patients with small native breasts who are candidates for nipple-sparing mastectomy can elect to undergo nipple and/or areolar reduction. In this technique, a diamond-shaped wedge of areola and nipple papule is fashioned to reduce the diameter of both the areola and nipple papule. This can be extended onto the papule to include and reduce the height or projection of the papule itself. The planned area is excised using a 15 blade is accompanied by subcutaneous tissue reduction below the papule to reduce the width and projection. The nipple is then closed in a simple interrupted fashion with absorbable suture such as chromic suture. The resected tissue is sent to pathology for analysis. This procedure can be offered as a revision in the operating room in conjunction with liposuction and fat grafting to enhance the chest contour or performed under local anesthesia in a clinic procedure room.

#### 2.1.3. Chest Wall Contouring: Liposuction Versus Soft Tissue Reduction and Fat Grafting

**Figure 4:**
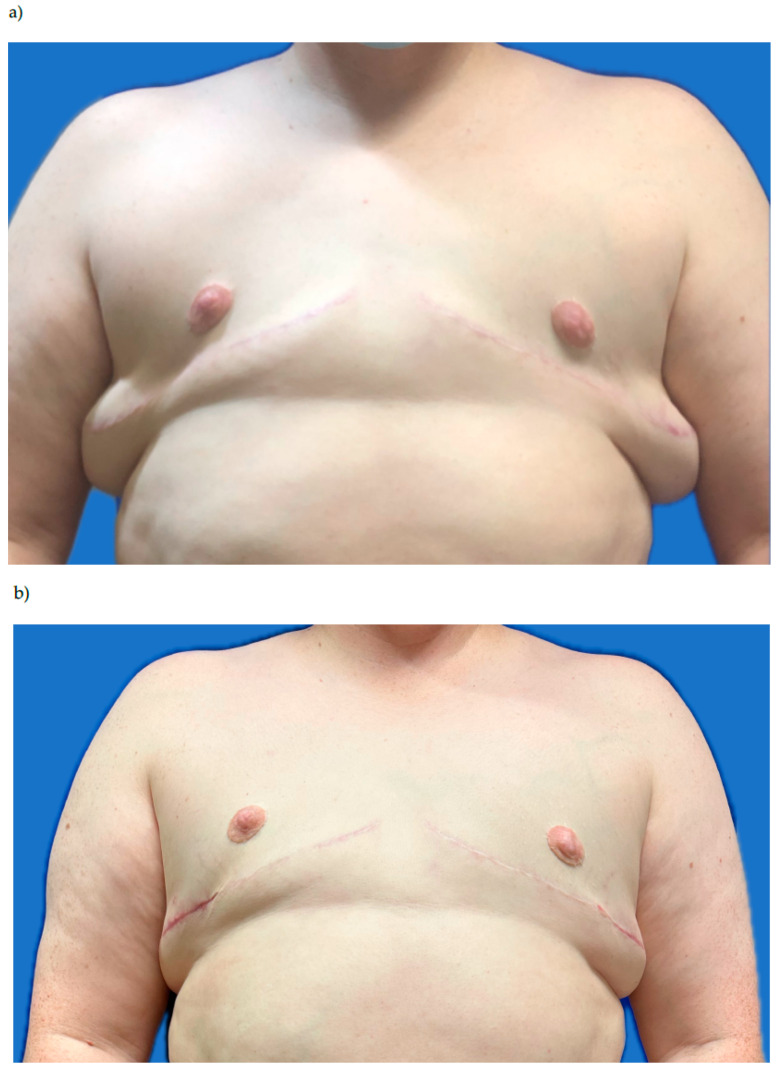
(**a**) Oncologic gender-affirming mastectomy with free nipple graft (**b**). After revision surgery with lateral chest liposuction, fat grafting to the anterior chest, and resection of excess lateral skin.

Compared to a standard gender-affirming mastectomy, an oncologic gender-affirming mastectomy has more volume resected with thinner skin flaps that can lead to more obvious excess lateral chest skin and subcutaneous fat. To enhance the overall contour of the chest and smoothen the transition of the anterior to lateral chest, we offer a mix of liposuction of the lateral chest wall versus tissue reduction to achieve an aesthetically favorable outcome (Figure 4). Counseling patients that revision surgery is possible and more common than in a traditional GAM is important. Typically, donor sites for liposuction and fat-grafting are similar to those of other breast reconstruction patients and include the abdomen, lateral chest, and flanks. A patient may need more than one round of liposuction and fat grafting to address contour abnormalities; this can be performed serially based on the patient’s body frame and desired outcome.

#### 2.1.4. Goldilocks Procedure

**Figure 5:**
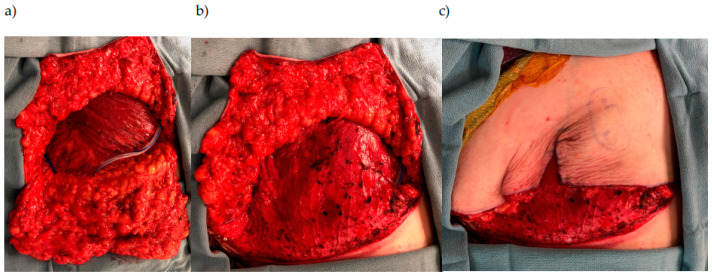
Goldilocks reconstruction (**a**) mastectomy with inferior dermal flap flipped down with drain placed between inferior dermal flap and pectoralis muscle. (**b**) Inferior skin flap de-epithelialized and rotated superiorly to cover the pectoralis muscle. (**c**) Superior skin over the inferior dermal flap.

For patients with larger ptotic breasts or those who desire more chest wall fullness to align with their non-binary identity, the Goldilocks procedure with IMF obliteration is a good option. A Goldilocks mastectomy utilizes the inferior chest dermal flap to create a small amount of fullness and contour to the chest. This is achieved by de-epithelializing the inferior mastectomy skin below the superior marking down to the IMF. For chest masculinization purposes, the dissection is carried inferiorly to completely obliterate the IMF, unlike a cis-female Goldilocks approach. However, in both surgeries, the inferior dermal flap is advanced superiorly. Medial and lateral back cuts along the inferior aspect of the inferior dermal flap are performed to advance the tissue centrally. The inferior tissue is sutured to the pectoralis muscle to maintain its position. The perfusion and blood supply of the dermal flap is assessed by evaluating for dermal bleeding—and, if necessary—perfusion can be assessed with SPY™ Elite Fluorescence Imaging and indocyanine green. All non-perfused or poorly perfused areas of the dermal flap should be resected prior to closure. The superior mastectomy flap is then draped over the inferior dermal flap and sutured to the inferior border of the dermal flap with lateral drain placement to avoid seroma formation (Figure 5).

#### 2.1.5. Nipple-Sparing Mastectomy Candidates and Staged Reconstruction

From an oncologic standpoint, NSM candidates are patients with high-risk mutations, early-stage malignancy, or locally advanced cancers without nipple involvement. It is important to note that guidelines vary on the criteria for ideal candidacy of NSM; however, the literature references small, non-ptotic breasts in non-smokers to preserve blood supply and reduce the risk of nipple necrosis [10,11]. In our case series, NSM was carried out via periareolar incision or inframammary fold incision with no reconstruction at the time of mastectomy. Both patients underwent a staged reconstruction under general anesthesia that included nipple reduction and chest contouring utilizing liposuction and fat grafting 3 months following mastectomy.

#### 2.1.6. Periareolar Mastectomy with Staged Reconstruction

Periareolar incisions can be utilized for NSM for patients with small, non-ptotic breasts that have good skin elasticity and who prefer to avoid transverse incisions on their chest. While these approaches result in smaller, less noticeable incisions, it is limited to patients who meet the above criteria. Nipple reduction and other procedures to improve chest contour cannot be performed simultaneously and thus are completed in a staged fashion.

**Figure 6:**
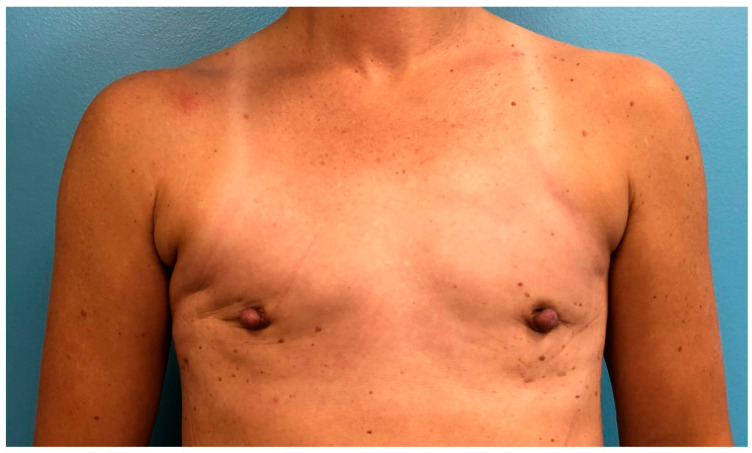
Nipple-sparing mastectomy with superior periareolar incision with small lateral extension, after reconstruction with nipple reduction, liposuction, and fat grafting for chest contour.

In a periareolar mastectomy, a semilunar periareolar incision is made around half of the nipple border, with small lateral extension as needed, to core out the breast parenchyma via a smaller, discrete incision (Figure 6). Similar to the double-incision mastectomies and Goldilocks technique, the IMF is purposely obliterated to improve chest contour. The patient is then sent home with continuous compression and bilateral drains to help the soft tissue contract down to the chest wall. Once post-operative swelling subsides and the incisions have healed, the patient is assessed for planned staged reconstruction with nipple and/or areola reduction, liposuction, and fat grafting.

## 3. Results

### 3.1. Demographics

A total of eight AFAB patients were included in this study, all of whom requested chest masculinization or non-binary reconstruction on their initial visit with their plastic surgeon. The mean age at the time of mastectomy was 35.13 years (SD = 8.04 years). The mean BMI was 29.88 (SD = 6.40). The mean total bilateral resection weight was 1336.8 g (SD = 879.2 g). All patients had a minimum of 12 months of post-operative follow-up, with a median follow-up of 40 months (mean of 41.4 months; range of 20–63 months). Half (*N* = 4) of the cohort was diagnosed with invasive ductal carcinoma (IDC) prior to surgery (two left breasts, two right breasts). The remaining patients were at high risk of developing breast cancer due to genetic predispositions and/or strong family history. One (12.5%) patient underwent neoadjuvant chemotherapy and adjuvant radiation, one patient received adjuvant endocrine therapy, and two patients did not require any neoadjuvant or adjuvant therapy. Regarding hormone therapy, three (37.5%) patients were on testosterone and continued hormone replacement therapy (HRT) post-mastectomy, and four (50%) patients were on menstrual suppression. Patient demographics, comorbidities, and oncologic status can be found in Table 1.

Of the eight patients who underwent OGAM, two patients underwent NSM (25%), two (25%) patients underwent SSM, and four (50%) patients underwent simple mastectomy. Of this cohort, the two (25%) NSM patients underwent a planned staged reconstruction in which they had a separate reconstructive surgery for chest masculinization with nipple reduction, liposuction, and fat grafting following recovery from their index oncologic mastectomy. Six (75%) patients underwent concurrent reconstruction at the time of the oncologic mastectomy. This included four (50%) patients who underwent double-incision GAM with FNG at the time of mastectomy, while two (25%) patients underwent goldilocks with or without FNG at the time of mastectomy. Secondary procedures under general anesthesia, such as liposuction and fat grafting, were utilized in four patients (50%), twice for planned staged chest masculinization and twice for revision surgery of OGAM and FNG. Direct liposuction and tissue excision were performed once for revision surgery following Goldilocks reconstruction. Perioperative and revision surgery data can be found in Table 2.

### 3.2. Clinical Outcomes

Of the eight total cases, one (12.5%) patient experienced a post-operative complication. This patient underwent bilateral simple mastectomy with concurrent Goldilocks reconstruction without FNG and experienced delayed wound healing after developing a surgical site infection with wound dehiscence. The patient was admitted for initiation of IV antibiotics, and cultures from the surgical site returned positive for Proteus mirabilis. The patient was discharged home two days later with a seven-day course of ciprofloxacin and local wound care. The remaining course of recovery was uneventful for this patient.

Three (37.5%) individuals underwent secondary revision surgery, which included excess lateral soft tissue removal along with bra line liposuction (Table 2). No patients in the cohort sought out reversal of gender-affirming surgery. Additionally, there were no significant associations between complications and/or revisions with hormone therapy exposure, endocrine therapy exposure, mastectomy technique, or reconstructive technique. Post-operative complications can be found in Table 3.

## 4. Discussion

It is standard practice that cisgender females who are diagnosed with breast cancer or at high risk for breast cancer have a consultation with both a breast and plastic surgeon. Currently, it is variable if TGD patients who require an oncologic lumpectomy or mastectomy but do not desire traditional oncoplastic, implant-based, or autologous breast reconstruction are offered referrals to plastic surgeons for consultation to discuss OGAM options. TGD patients may benefit from consulting with a plastic surgeon to discuss procedures that can concur with their oncologic mastectomy to help address their gender dysphoria and desire for chest masculinization or non-binary reconstruction [8,12,13]. The recent breast oncology and reconstructive literature has emphasized the importance of balancing oncologic safety with reconstructive and aesthetic outcomes—particularly in nipple-sparing and individualized surgical approaches—reinforcing the role of multidisciplinary, patient-centered decision-making frameworks that are directly applicable to OGAM for TGD patients [14,15].

To our knowledge, this case series represents the first and largest cohort to date discussing a wide variety of surgical techniques and outcomes for TGD patients undergoing OGAM. We report the results of eight patients who underwent OGAM at our institution from 2019 to 2023. Indications for mastectomy included four (50%) patients with a diagnosis of invasive ductal carcinoma and four (50%) patients with a strong family history of breast cancer or genetic predisposition to breast cancer seeking risk-reducing mastectomies (e.g., BRCA mutations, MSH mutations). Six (75%) patients were primary surgical candidates, while one patient required neoadjuvant chemotherapy and post-mastectomy radiation, and another received adjuvant endocrine therapy. Mastectomy options included NSM, SSM, and simple mastectomy. Reconstructive techniques included double-incision OGAM with or without nipple grafting, Goldilocks procedure with or without FNG, secondary nipple and/or areola reduction, liposuction, fat grafting, and/or excision of excess skin and soft tissue for chest contouring. These procedures can be offered to patients in a concurrent or staged manner, as seen in our patient cohort. Compared to a standard gender-affirming mastectomy, an OGAM results in thinner superior mastectomy skin flaps frequently requiring IMF obliteration and thinning of the caudal aspect of the inferior mastectomy flap to achieve the appropriate contour between the chest and abdomen. Counseling patients ahead of time that liposuction and fat grafting may be needed to improve the chest contour is paramount, as evidence that 50% of our double-incision mastectomy patients had revision surgery to improve overall chest aesthetics.

One (12.5%) patient experienced a surgical site infection and wound dehiscence four days status post OGAM with the Goldilocks technique. This patient had several comorbidities that increased their risk of infection, including obesity (BMI = 41) and long-term use of immunosuppressive medication for the treatment of Crohn’s disease. As seen in the literature, prior work has shown BMI to be an independent risk factor for post-mastectomy complications (all-cause and wound complications) in both cisgender and transgender patients [16]. No complications were noted in the cohort at one-year post-operation, including requests for reversal of gender-affirming surgery. Additionally, there have been no reported cases of cancer recurrence within our cohort to date. Our work documents three patients continuing their HRT during cancer treatment without observed reconstructive complications. Currently, the associations between breast cancer and HRT are not well known. While some speculate that testosterone may help reduce breast cancer risk and therefore incidence of breast cancer in TGD patients, there is inconclusive evidence between elevated androgen levels and breast cancer in AFAB patients [1,9,17,18]. It is important to state that, given our small cohort size, these findings are descriptive and should be interpreted cautiously, as this series was not powered to assess associations between hormone therapy and surgical outcomes.

There is a paucity of information on breast cancer in the AFAB TGD population, including surgical techniques and surgical outcomes [1]. The previous literature has documented a limited number of cases of OGAM in which solely the standard double-incision technique was utilized, with or without FNG [1,9]. Our series expands upon this knowledge by demonstrating reconstructive techniques other than the double-incision GAM and FNG that can be offered to patients seeking OGAM. These techniques include Goldilocks, nipple reduction, liposuction, fat grafting, and excess tissue resection either at the time of index surgery or staged to improve chest contour. Our case series further supports the previous limited literature that OGAM for cancer treatment is a safe option [1]. It is important to emphasize the need for interdisciplinary care and ongoing advocacy for the marginalized TGD population. Both breast and plastic reconstructive surgeons play a unique role in educating these patients about the options available to address both breast cancer and desires for chest masculinization or non-binary reconstruction, while also embracing the responsibility to provide ongoing support and reduce barriers to care. The coordination in care allows for multi-disciplinary counseling, planned patient markings, and unified care plans for patients to provide timely and appropriate care.

### Limitations and Future Directions

Limitations inherent to case series are expected, given the small sample size, retrospective design, and limited ability to generalize findings to the broader TGD and oncologic gender-affirming mastectomy patients or definitive care recommendations. Additionally, our review does not include patient-reported outcomes or validated satisfaction instruments, limiting our ability to assess satisfaction with chest contour, alignment with gender identity, or psychosocial well-being following OGAM. Given the importance of patient-reported outcomes in TGD surgical care, this represents an important area for future investigation. Future directions should include studies with increased patient numbers, longer follow-up periods, patient-reported outcomes measures, and multi-center reporting. This would help obtain more data on the variety of treatment options and patient outcomes. Additionally, assessing patient decisional regret and satisfaction scores could inform future quality improvement efforts regarding patient education and setting expectations.

## 5. Conclusions

This small case series highlights the importance of a multidisciplinary, personalized approach for AFAB TGD individuals with breast cancer, or at high risk for breast cancer, seeking OGAM. We reviewed various reconstructive options provided to TGD patients at our facility, demonstrating the array of safe oncologic and reconstructive techniques available for this population. This emphasized the need for ongoing collaboration between breast and plastic surgeons and community awareness regarding OGAM and non-binary reconstruction options. Further large-scale studies highlighting various techniques and prospective studies assessing patient-reported outcomes would be valuable additions to the literature.

## Figures and Tables

**Table 1:** Demographics.

Patient Number	1	2	3	4	5	6	7	8
Age	42	41	30	26	43	22	37	40
Race	White	White	White	White	White	White	American Indian/Alaskan	Asian
Ethnicity	Non-Hispanic	Non-Hispanic	Non-Hispanic	Non-Hispanic	Non-Hispanic	Non-Hispanic	Hispanic	----
Gender	GF	GF	GQ	GQ	NB	NB	Other	NB
Sexual orientation	Lesbian/Gay	Queer	Bisexual	Queer	Gay	Bisexual	Bisexual	----
BMI	24	27	41	26	34	28	36	23
Insurance type	Private	Private	Private	Private	Private	Private	Medicaid	Medicaid
Smoking history	Never	Former	Never	Never	Never	Never	Never	Never
Diabetes history	N	Prediabetic	N	N	Prediabetic	Prediabetic	N	N
Hormone replacement therapy (testosterone)	N	N	Y	N	Y	N	Y	N
Menstrual suppression	Y	N	Y	N	Y	Y	N	N
Cancer type	IDC	----	----	----	----	IDC	IDC	IDC
BC predisposition	----	Li-Fraumeni	BCRA status	Strong family history	BRCA status, MSH3 mutation	----	----	----
Prophylactic case	N	Y	Y	Y	Y	N	N	N
Laterality	L	----	----	----	---	R	R	L
Breast quadrant	1	----	----	----	----	2	4	1
ER	+	---	----	----	----	−	+	+
PR	+	---	----	----	----	−	+	+
HER	-	---	----	----	----	−	−	−
Lymph node involvement	N	---	----	----	----	Y	N	N
Oncologic staging	pT2N0M0	---	----	----	----	cT2N3bM0	cT1cN0	pT2N0M0

GF—gender fluid; NB—non-binary; GQ—gender queer; N—no; Y—yes; L—left; R—right; IDC—invasive ductal carcinoma.

**Table 2:** Surgical information.

Patient Number	1	2	3	4	5	6	7	8
Oncologic procedure	Periareolar NSM	SM	SSM	SSM	SM	SM	SM	Periareolar NSM
Oncologic procedure laterality	Both	Both	Both	Both	Both	Both	Both	Both
Resection weight left (g)	177	449	1527	1020	532	428	893	269
Resection weight right (g)	181	411	1411	972	624	434	1065	301
Adjuvant chemo	N	-----	-----	-----	-----	Y	N	N
Radiation	N	-----	-----	-----	-----	Y	N	N
Endocrine therapy	Y	-----	-----	-----	-----	N	N	N
Reconstructive technique	Nipple reduction + FG	GAM, flat closure + FNG	Goldilocks	Goldilocks, FNG	GAM, flat closure, FNG	GAM, flat closure, FNG	GAM, flat closure, FNG	Nipple reduction, FG
Reconstructive staging	Staged nipple reduction, FG.	concurrent GAM + FNG	concurrent Goldilocks	concurrent GAM + FNG	concurrent GAM + FNG	concurrent GAM + FNG	concurrent Goldilocks + FNG	Staged nipple reduction, FG.
Revision info	no revision	no revision	liposuction and tissue excision	no revision	liposuction of the lateral chest wall, fat grafting to inferior chest, excision of dog ear	liposuction of the lateral chest wall, fat grafting to inferior chest, excision of dog ear	no revision	no revision
Fat grafting (initial reconstruction or planned revision)	Y	N	N	N	Y	Y	N	Y

NSM—nipple-sparing mastectomy, SM—simple mastectomy, SSM—skin-sparing mastectomy; N—no; Y—yes; L—left; R—right; FG—fat grafting; FNG—free nipple graft; GAM—gender-affirming mastectomy.

**Table 3:** Complications.

Patient Number	1	2	3	4	5	6	7	8
Complications	none	none	SSI, readmission, wound dehiscence	none	none	none	none	none

SSI—surgical site infection.

## Data Availability

The original contributions presented in this study are included in the article material. Further inquiries may be directed to the corresponding author.

## Abbreviations

The following abbreviations are used in this manuscript:

AFAB	Assigned Female at Birth
TGD	Transgender or Gender Diverse
(O)GAM	(Oncologic) Gender-Affirming Mastectomy
SM	Simple Mastectomy
SSM	Skin-Sparing Mastectomy
NSM	Nipple-Sparing Mastectomy
FNG	Free Nipple Grafting
IMF	Inframammary Fold
HRT	Hormone Replacement Therapy
IDC	Invasive Ductal Carcinoma
